# Supplementary material for: A Dyadic Approach to Cancer Care: Examining the Feasibility and Preliminary Effectiveness of a Partner-Based Exercise Intervention for Caregivers and Their Care Recipients
Source: Int J Environ Res Public Health. 2025 Dec 31;23(1):56. doi: 10.3390/ijerph23010056 (PMC12840962; doi:10.3390/ijerph23010056)
Supplement: Supplementary file 1 [file ijerph-23-00056-s001.zip › ijerph-4024155-supplementary.pdf]

Supplementary Table S1. Number of participants in each Role:Time cell.

	Patients		Caregivers	
**Outcome**	**Baseline**	**Post-Intervention**	**Baseline**	**Post-Intervention**
**ED5Q5L Outcomes**				
Anxiety/Depression Subscale	21	19	21	21
Mobility Subscale	21	19	21	21
Pain/Discomfort Subscale	21	19	21	21
Self-care Subscale	21	19	21	21
Usual Activities Subscale	21	19	21	21
Health Visual Analog Scale	21	19	21	21
Index Score	21	19	21	21
**PSQI Outcomes**				
PSQI Total Score	21	19	21	21
**Physical Activity Outcomes**				
Mild Physical Activity	21	19	21	21
Moderate Physical Activity	21	19	21	21
Strenuous Physical Activity	21	19	21	21
Total Physical Activity	21	19	21	21
**RAND-36 Outcomes**				
Physical Functioning Subscale	21	19	21	21
Limitations Physical Health Subscale	21	19	21	21
Limitations Emotional Subscale	21	19	21	21
Energy/Fatigue Subscale	21	19	21	21
Emotional Wellbeing Subscale	21	19	21	21
Social Functioning Subscale	21	19	21	21
Pain Subscale	21	19	21	21
General Health Subscale	21	19	21	21
**DASS Outcomes**				
DASS Total Score	21	19	21	21
Depression Subscale	21	19	21	21
Anxiety Subscale	21	19	21	21
Stress Subscale	21	19	21	21

**Caregiver Burden**				
Burden in Relationship	NA	NA	20	21
Loss of Control	NA	NA	20	21
Roll Strain	NA	NA	20	21
**Fitness Assessment**	NA	NA		
Heart Rate	21	19	21	19
Resting Systolic Blood Pressure	21	19	21	19
Resting Diastolic Blood Pressure	21	19	21	19
6 Minute Walk Test Laps	21	17	21	19
6 Minute Walk Test Meters	21	17	21	19
RPE For 6 Minute Walk Test	21	17	21	19
Combined Grip Strength	20	19	20	19
30s Sit-to-Stand	21	18	21	19
Flexometer	19	16	19	19
BMI	21	19	21	19
Waist Circumference	21	19	21	19
Hip Circumference	21	19	21	19
